# The Road to Gold: Training and Peaking Characteristics in the Year Prior to a Gold Medal Endurance Performance

**DOI:** 10.1371/journal.pone.0101796

**Published:** 2014-07-14

**Authors:** Espen Tønnessen, Øystein Sylta, Thomas A. Haugen, Erlend Hem, Ida S. Svendsen, Stephen Seiler

**Affiliations:** 1 The Norwegian Olympic Federation, Oslo, Norway; 2 Faculty of Health and Sport Sciences, University of Agder, Kristiansand, Norway; 3 School of Sport, Exercise and Health Sciences, Loughborough University, Leicestershire, United Kingdom; University of Maribor, Slovenia

## Abstract

**Purpose:**

To describe training variations across the annual cycle in Olympic and World Champion endurance athletes, and determine whether these athletes used tapering strategies in line with recommendations in the literature.

**Methods:**

Eleven elite XC skiers and biathletes (4 male; 28±1 yr, 85±5 mL. min^−1^. kg^−1^ 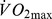, 7 female, 25±4 yr, 73±3 mL. min^−1^. kg^−1^ 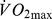) reported one year of day-to-day training leading up to the most successful competition of their career. Training data were divided into periodization and peaking phases and distributed into training forms, intensity zones and endurance activity forms.

**Results:**

Athletes trained ∼800 h/500 sessions.year^−1^, including ∼500 h. year^−1^ of sport-specific training. Ninety-four percent of all training was executed as aerobic endurance training. Of this, ∼90% was low intensity training (LIT, below the first lactate threshold) and 10% high intensity training (HIT, above the first lactate threshold) by time. Categorically, 23% of training sessions were characterized as HIT with primary portions executed at or above the first lactate turn point. Training volume and specificity distribution conformed to a traditional periodization model, but absolute volume of HIT remained stable across phases. However, HIT training patterns tended to become more polarized in the competition phase. Training volume, frequency and intensity remained unchanged from pre-peaking to peaking period, but there was a 32±15% (P<.01) volume reduction from the preparation period to peaking phase.

**Conclusions:**

The annual training data for these Olympic and World champion XC skiers and biathletes conforms to previously reported training patterns of elite endurance athletes. During the competition phase, training became more sport-specific, with 92% performed as XC skiing. However, they did not follow suggested tapering practice derived from short-term experimental studies. Only three out of 11 athletes took a rest day during the final 5 days prior to their most successful competition.

## Introduction

Winning a gold medal in a major international championship requires not only outstanding athletic ability and long-term training progression, but also that the athlete achieves peak performance at the right time. In recent years, increased attention has been given to quantifying the training characteristics of elite endurance athletes [1]–[3] and this information has provided a fruitful foundation for hypothesis testing regarding training load and physiological adaptation. At the same time, a strong knowledge base has developed regarding best practice for the tapering and peaking process, based largely on experimental interventions [4]–[6]. However, studies linking the characteristics of the long-term training process to those of the short term pre-peaking and peaking process are lacking.

Recently, a number of descriptive studies, both retrospective and prospective, have been published on the training characteristics of athletes from endurance sports such as running [7]–[12], cycling [13]–[14], XC skiing [15]–[17], swimming [18]–[19], rowing [20]–[21], triathlon [22]–[23], speed skating [24]–[25] and kayaking [26]. Training load variables such as volume, frequency and intensity distribution appear to play an interactive role in maximizing physical capacity and performance [27]. Depending on the specific muscular loading characteristics of the sport, athletes typically train 500 h (distance running) [7], [8], [11], [12], [28], [29] to well in excess of 1000 h per year (rowing, swimming, cycling, triathlon) [13]–[14], [18]–[23] performed during 400–800 annual training sessions [11]–[12], [15]–[17], [23], in order to reach an internationally elite level. When examining the intensity distribution of this large training volume, a number of studies across a broad range of sports converge on the finding that 75–90% of all endurance training time is performed as low intensity training (LIT, below the first lactate turn point) for athletes training >500 h per year. The remaining 10–25% is comprised of high intensity training (HIT) performed above the first lactate turn point [7]–[8], [14]–[15], [22]–[23]. This approximate “80–20” distribution between low and high intensity training among high-level performers is a robust finding [3], even if mechanistic explanations for its ubiquity remain speculative. Furthermore, the “best practice” magnitude and distribution of HIT remains unclear. In addition, the training-dose response relationship has a significant individual variation component, which further complicates the generalizability of the picture. While the influx of descriptive data on the training of elite endurance athletes has informed training practice, and stimulated new experimental studies, methodological compromises are inherent to the challenge of measuring behavior reliably and precisely in highly selected groups. Available descriptive studies typically only present data over a shorter time frame [7]–[8], [12], at a sub-elite level [9], [14]–[15], [17], [21]–[22] or as single case studies [11], [23], [28]–[29]. Accuracy in training monitoring is also unclear, due to weaknesses in methods such as questionnaires [10] or compilation and analysis of data that are in part or completely based on training plans instead of strict quantification of actual training performed [7]–[8], [20]. Limited data currently exist on the long-term training of highly trained and elite athletes, based on accurate training monitoring [30].

Short-term training manipulations to achieve peaking for optimal sports performance have been investigated for more than 20 years. A synthesis of studies on well-trained athletes from a variety of different sports has shown a performance improvement of up to 3%, providing the final 4–28 days of training are executed correctly [5]–[6], [31]–[33]. All other things being equal, a well-executed peaking phase can therefore dramatically increase the odds of winning a gold medal at a championship event for an individual athlete with finalist potential. In the research literature, the peaking process is typically divided into two phases: a pre-tapering phase, and the final taper period culminating with the intended competition. The aim of the pre-tapering phase is to stimulate a controlled “over-reaching” state and elicit a super-compensatory adaptive response in the following taper. Experimentally, the optimal duration of the actual taper depends on the training executed in the pre-taper [6], [33]–[34]. The taper is initiated approximately 14 days prior to the desired peak performance, and the aim of this phase is to facilitate regeneration and reduce fatigue, while maintaining or increasing fitness and technical/psychological readiness in order to mobilize maximal performance in competition [4]–[6], [35]. The optimal training volume, frequency and intensity in each phase is debated [4]–[6], [31], [35], but a reduction in training load of between 41–60% from pre-taper to taper has been recommended. This reduction appears to be best achieved via reduced training duration per session, while maintaining session frequency [5]. Maintaining training intensity is considered to be a key factor in a successful peaking regime, and it is therefore recommended that the frequency of HIT sessions be maintained during the taper [36]–[39]. Despite 20 years of research on tapering and peaking for athletic performance, we are unaware of any study that has successfully quantified the “real life” peaking strategies of athletes achieving ultimate success in Olympic Games or World Championship events. It is therefore unclear how the models developed from controlled experimental studies are translated to the actual peaking practices of elite endurance athletes. Furthermore, few studies havelinked peaking strategies to annual training characteristics and the competitive season of elite athletes [30].

The current study therefore aimed to: 1) present highly accurate day-to-day annual training data from a cohort of endurance athletes that all won Olympic or World Championship gold medals and, 2) quantify and examine relationships between annual training and peaking characteristics in these athletes.

## Methods

### Subjects

Four male and seven female former and current Norwegian elite XC skiers and biathletes were included in the study (Table 1). All athletes had won a least one individual Olympic or World Championship senior gold medal during their career. In total, included males had won 41 (5–26) and females 25 (1–9) gold medals (includes both individual and relays from 1985 through 2011). In addition, included athletes had systematically and accurately recorded their day-to-day training in detail from junior through to senior level. In the current study, we have analyzed and reported the year specifically leading up to their most successful competition at senior level. The regional ethics committee of Southern Norway reviewed the study and concluded that, due to the nature of the investigation, it did not require their approval. The study was therefore submitted to and approved by the Norwegian Social Science Data Services (NSD), and all athletes gave their oral and written informed consent prior to study participation.

**Table 1:** General characteristics of athletes included in the study.

Subject	Gender	Age	Height	Weight	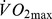 (ml^.^kg^−1^.min^−1^)	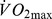 (l.min^−1^)
1	M	28	180	77	92.5	7.13
2	M	26	190	82	81.9	6.73
3	M	29	189	83	84.8	7.07
4	M	28	179	66	81.2	5.25
5	F	23	172	55	72.9	3.90
6	F	23	176	63	73.6	4.64
7	F	29	173	63	76.6	4.81
8	F	20	175	69	70.4	4.83
9	F	28	166	61	69.1	4.24
10	F	22	162	51	76.0	3.93
11	F	30	169	64	71.4	4.60
Mean ± SD, Male		28±1	185±6	77±8	85.1±5.2	6.5±0.9
Mean ± SD, Female		25±4	170±5	61±6	72.9±2.8	4.4±0.4

Values are reported from the analyzed year in the current study.

### Physiological testing

All athletes underwent regular physiological testing during their career. The test values presented in Table 1 represent the highest result achieved during the analyzed year. There were no physiological tests performed during the competition period, and the presented results therefore represent tests from October or November, while Olympic or World Championship events were typically held in February-March. All physiological testing was conducted at the Norwegian Olympic training center. 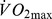 testing was performed as running at 10.5% inclination on a motorized treadmill (Woodway Gmbh, Weil am Rhein, Germany) calibrated for speed and incline. The procedure started with an extensive warm-up sequence, followed by a stepwise increase in running velocity every minute thereafter until volitional exhaustion, normally occurring after 4–6 minutes. Starting velocity for all athletes corresponded to 85–90% of 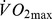. The increase was 1 km.h^−1^.min^−1^, and the last velocity step was held for at least 1 min. The test was terminated before voluntary exhaustion if the 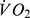
values leveled off or decreased despite increasing workload and ventilation, in addition to respiratory exchange ratio (RER) >1.10. 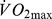 was defined as the highest average of two consecutive 30 s measurements. Oxygen uptake was measured using EOS Sprint (Jaeger-Toennis, Wurtzburg, Germany) until 2002, after which an Oxycon Pro (Jaeger-Toennis, Wurtzburg, Germany) metabolic test system was used. An internal comparison between the two analyzers was conducted during the transition in 2002 and showed identical regression lines for the treadmill running velocity –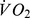 relationship with both systems. Primarily two exercise physiologists supervised all testing during the entire period.

### Training monitoring

**Figure 1:**
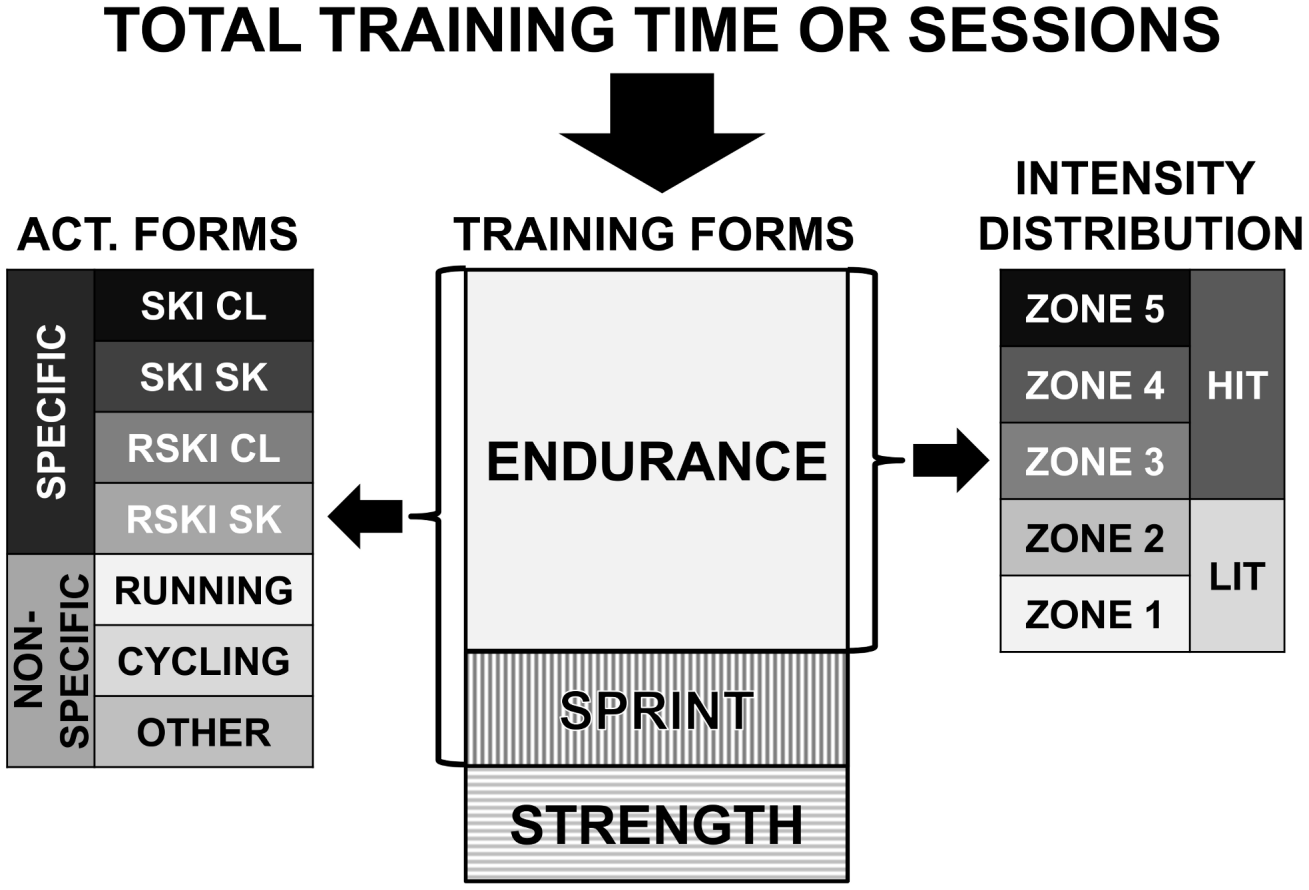
Training distribution methods. Total training time was divided into training forms (endurance, sprint and strength). Endurance time and frequency were further distributed into 5 intensity zones in line with table 2. Zones 1–2 are LIT and zones 3–5 are HIT. Endurance and sprint time together were divided into activity forms. Ski and roller ski were classified as specific, and running, cycling or other as non-specific activity forms.

Athletes included in the study recorded their day-to-day training during their most successful year in paper diaries designed by the Norwegian Ski Association [40]–[41], the Norwegian Biathlon Association [42] or, since ∼2005, in the digital version developed by the Norwegian Olympic Federation (OLT). The training recorded for each session included total training time distributed across training form (strength, endurance, sprint), activity form (skiing, roller-skiing, running, cycling etc.), and intensity zone, as well as specific comments regarding session details. All paper training diaries were transferred session by session to digital format by persons from the current research group. Total training time and frequency of sessions was distributed in line with the structure in Figure 1. Digitized diary data was rigorously cross-checked for internal consistency among different training distribution breakdowns at the individual level. Internal consistency of digitized training records from all included athletes was ≥99%.

All the athletes included in the study used a 5 intensity zone model, where zones 1–2 are classified as LIT and zones 3–5 as HIT. The intensity scale presented in Table 2 represents average self-reported zone-cut offs from 29 elite XC-skiers from a previous study [43]. In the results section we have presented the data either in a binary model (LIT/HIT) or a 5-zone model were zones 1–2 are below the first lactate threshold (LT^1^), zone 3 between LT^1^ and LT^2^, and zones 4–5 above LT^2^ [3], [44]. The intensity distribution is classified both according to a time in training zone approach and a frequency based session goal approach (SG). These methods and the intensity zones cut-offs have been described in detail recently [43].

**Table 2:** The 5-zone, 3-zone, and binary intensity scales used in the current study.

Intensity Zone	Typical Blood lactate^A^ (mmol. L^−1^)	Typical Heart Rate (% max)	Three zone model	Binary model
5	>5.8	>94	>LT^2^	
4	3.7–5.7	89–93		HIT
3	2.1–3.6	84–88	LT^1^–LT^2^	
2	1.3–2.0	74–83		LIT
1	<1.2	54–73	<LT^1^	

A Measured with Lactate Pro LT-1710. Reference values presented are derived from the average self-reported zone-cut offs of 29 elite XC-skiers [43], and individual adjustments are required.

### Annual periodization phases and peaking model

General training data from the entire year are either presented as annual training characteristics or divided into different periodization phases as presented in Table 3. Peaking characteristics were quantified based on the final 6 weeks of training prior to the gold medal winning performance, as delineated in Table 3.

**Table 3:** Training phases in annual cycle, including peaking phases.

Period in annual training cycle	Duration
Preparation period (PP)	May-December
* Transition period*	*May*
* General preparation period (GP)*	*June-October*
* Specific preparation period (SP)*	*November-December*
Competition Period (CP)	January-March
* Pre-peaking phase*	*6*–*3 weeks before championship event*
* Peaking phase*	*Last 14 days before championship event*
Regeneration period	April

### Statistical analyses

All data in text, tables or figures are presented as mean ± standard deviation (SD) and/or range. Statistical comparisons between different periodization phases are focused on the general preparation period (GP), specific preparation period (SP) and competition period (CP) in addition to comparing the actual peaking phase with pre-peaking phase, GP and SP. Data were not normally distributed. Therefore each variable from the GP, SP and CP (overall, pre-peaking and peaking phase) was tested with a non-parametric Friedman test, followed by a post-hoc test (Wilcoxon Signed Rank) to locate statistical differences. Male and female athlete data are merged, as a Mann-Whitney U Test revealed no significant differences in any relevant variables across gender (data not shown). All figures and statistical analyses were performed using Microsoft Excel or SPSS 18.0 (SPSS Inc, Chicago, IL, USA) and statistical significance was accepted at the *P*<.05 level or Bonferroni adjusted alpha level.

## Results

### Annual training characteristics

Total training volume was 770±99 h (622–942) distributed across 470±68 sessions (375–585) throughout the gold medal year. Endurance training accounted for 94±3% of all training time with the remaining 5±2% composed of strength training and 1±1% ski sprint training. Time in training zone based intensity distribution showed that 91±1% of all endurance training time was executed as LIT (zone 1–2) and 9±1% as HIT (zone 3–5).

**Figure 2:**
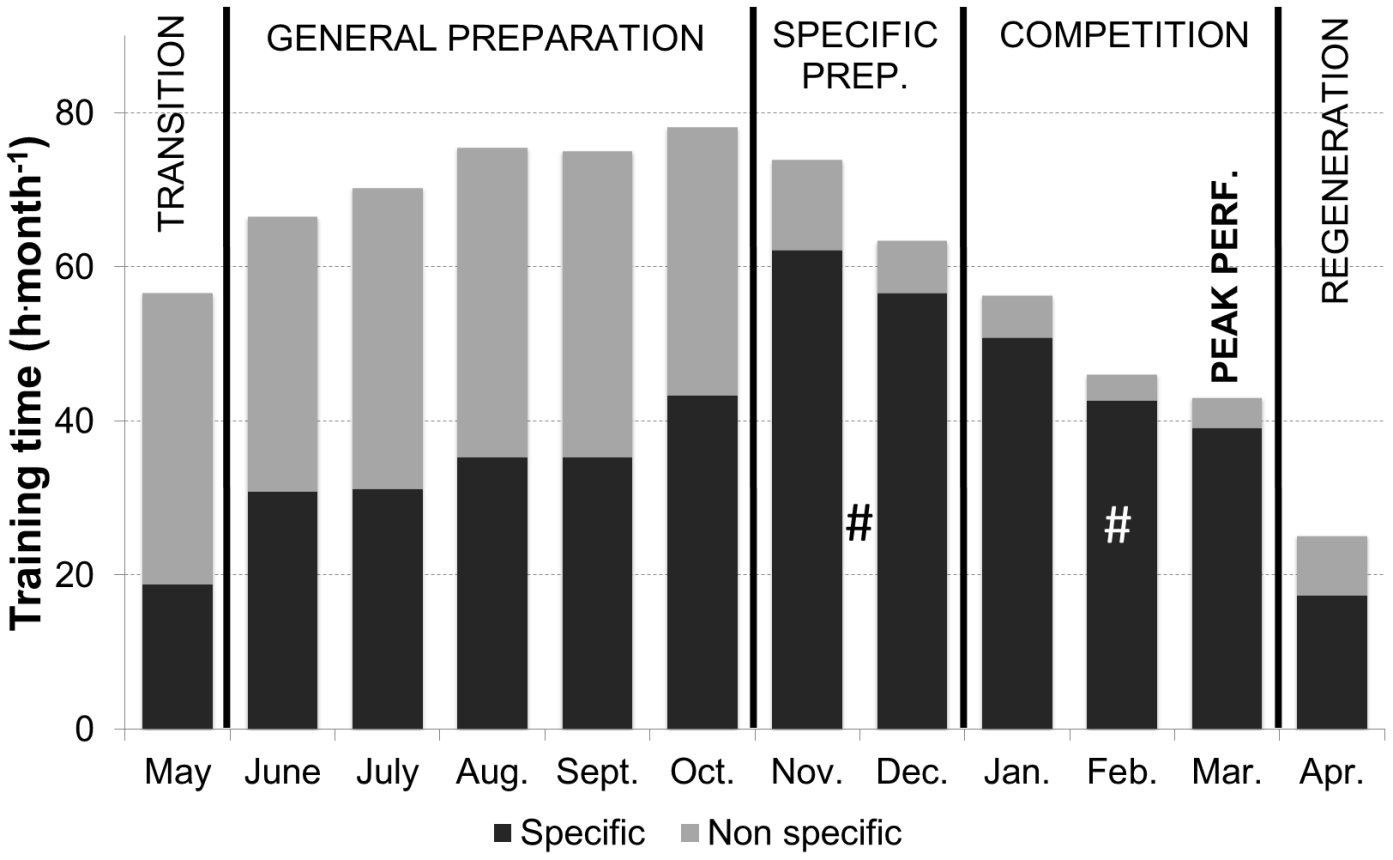
Annual organization of specific and non-specific activity forms. Endurance and sprint training time (h) distributed into specific (ski and roller ski) and non-specific (running, cycling and other) activity forms during each month and divided in phases. # Difference in specific training time vs. GP (*P*<.01).

Monthly training distribution of specific and non-specific activity forms during each training phase are presented in Figure 2. Endurance and sprint training was executed with sport-specific movement patterns (ski or roller ski) for 64±3% (465±56 h/min-max: 376–569 h) of total training time, with the remaining 36±3% (265±47 h/min-max: 196–337 h), composed of non-specific activity forms (running, cycling etc.) throughout the year. The proportion of sport-specific training increased significantly from GP (48±6%) to SP (87±8%) and CP (92±4%) (*P*<.01).

**Figure 3:**
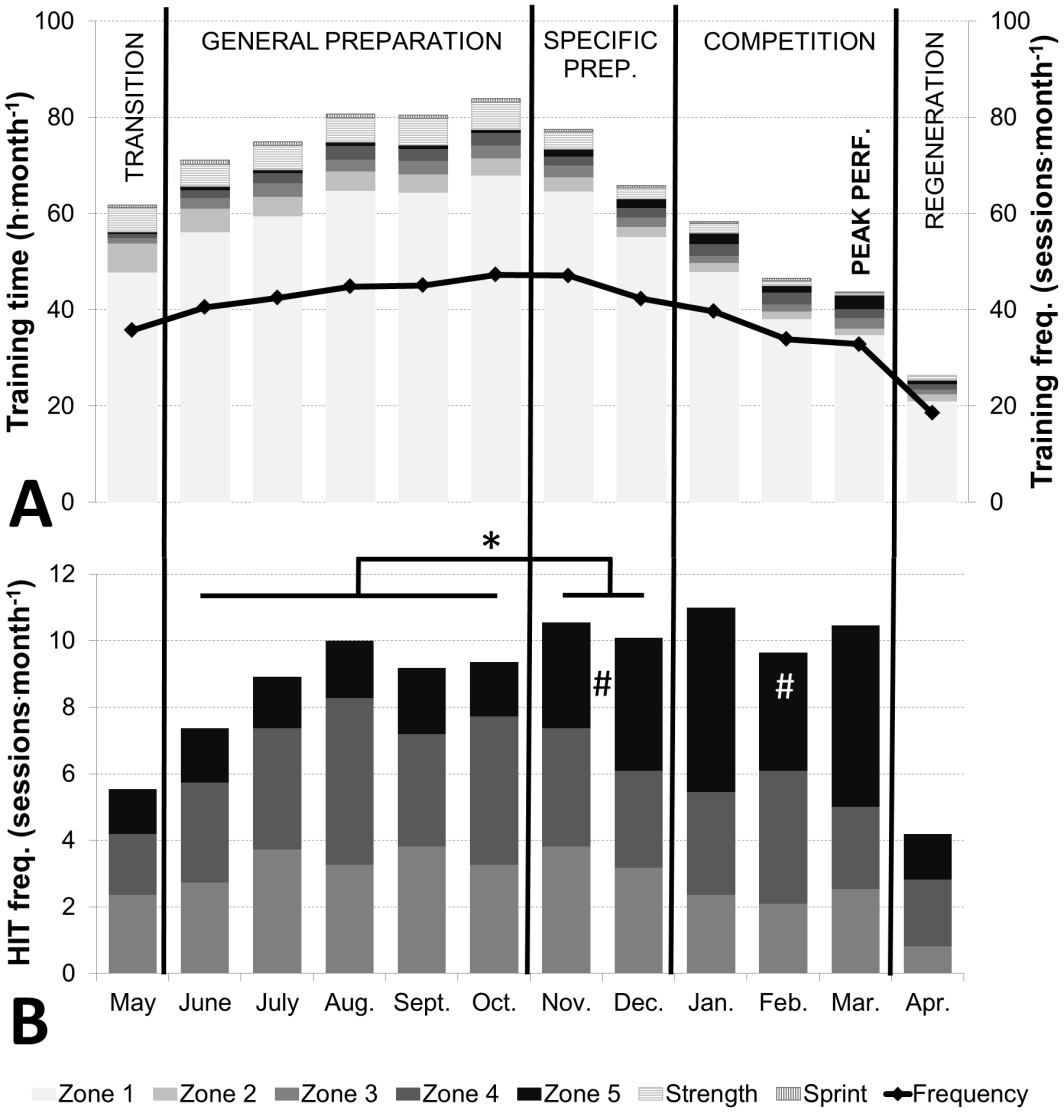
Annual training characteristics. A: Total training time (h) distributed into endurance training (zones 1–5), strength and sprint (bars, y-axis), and total training frequency (sessions) (line, z-axis) during each month and divided into phases. B: HIT frequency (sessions) distributed into zones 3, 4 and 5 (bars, y-axis) during each month and divided in phases. There was a statistically significant difference (P<.05) in total HIT sessions and zones 3, 4 and 5 respectively across the GP, SP and CP. Pairwise post-hoc tests showed: * Difference in total HIT sessions across phases (P<.01). # Difference between zone 5 sessions vs. GP (P<.01).

The distribution across all five intensity zones was: zone 1: 86.0±3.4%, zone 2: 5.3±3.0%, zone 3: 3.3±0.9%, zone 4: 3.3±1% and zone 5: 2.1±1.0%. When all endurance sessions were nominally categorized using the SG approach, the distribution was 77±2% LIT and 23±2% HIT (Figure 3, A). Weekly training patterns during each training phase are presented in Table 4.

**Table 4:** Weekly training patterns during different phases throughout the season.

				Competition phase			
*Weekly training patterns*	Transition phase	General preparation phase	Specific preparation phase	Overall	Pre-peaking	Peaking	Regeneration phase
**Total training:**							
**Training time (h.wk^−1^)**	13.9±5.3	17.9±2.5	16.5±2.4	11.6±2.2 ^#β^	13.5±3.1 ^#β^	12.1±2.4 ^#β^	6.1±3.7
**Tr. sessions^.^wk^−1^**	8.1±2.8	10.1±1.0	10.3±1.7	8.3±2.1 ^#β^	8.9±1.9 ^#β^	9.2±2.3	4.3±2.3
**Training forms:**							
**Endurance (h^.^wk^−1^)**	12.7±4.9	16.5±2.6	15.7±2.2	11.2±2.0 ^#β^	12.9±2.8 β	11.7±2.3 ^#β^	5.9±3.6
**Strength (h^.^wk^−1^)**	1.1±1.0	1.2±0.5	0.7±0.4 *	0.3±0.2 ^#β^	0.5±0.4 #	0.2±0.2 ^#β^	0.2±0.3
**Sprint (h^.^wk^−1^)**	0.1±0.1	0.2±0.2	0.1±0.1	0.1±0.1	0.1±0.1	0.2±0.2	0.0±0.0
**Intensity distribution:**							
**Zone 1 (h^.^wk^−1^)**	10.8±4.7	14.3±2.6	13.8±2.1	9.4±2.3 ^#β^	11.2±2.8 β	9.7±2.0 ^#β^	4.9±2.9
**Zone 2 (h^.^wk^−1^)**	1.3±0.9	0.9±0.6	0.6±0.5 *	0.4±0.3 #	0.4±0.5 #	0.6±0.8	0.3±0.5
**Zone 3 (h^.^wk^−1^)**	0.3±0.2	0.6±0.2	0.5±0.2	0.4±0.3	0.4±0.2 #	0.4±0.4	0.2±0.3
**Zone 4 (h^.^wk^−1^)**	0.2±0.2	0.5±0.2	0.4±0.3	0.5±0.3	0.5±0.4	0.7±0.4 β	0.3±0.3
**Zone 5 (h^.^wk^−1^)**	0.1±0.1	0.2±0.1	0.4±0.2 *	0.5±0.3	0.4±0.3	0.2±0.3	0.2±0.2
**Activity forms:**							
**Specific (h^.^wk^−1^)**	4.3±3.3	8.1±1.7	13.7±2.3 *	10.3±1.5 ^#β^	12.2±2.6 #	10.8±1.8 #	4.1±3.2
**Non-Specific (h^.^wk^−1^)**	8.5±4.6	8.6±1.5	2.1±1.2 *	1.0±0.6 ^#β^	0.8±0.6 ^#β^	1.0±1.0 ^#β^	1.8±1.5

Values are mean ± SD and represent training hours per week in different phases. A non-parametric Friedman test indicated that there was a statistically significant difference in all variables across the GP, SP and CP (P<.05 level). A pairwise post-hoc test (Wilcoxon Signed Rank) was used to determine whether there was a statistically significant difference between the GP, SP or CP, as well as pre-peaking and peaking phases (P<.01 level).

*P<.01, GP vs. SP;

# P<.01, GP vs. CP, pre-peaking or peaking;

β P<.01, SP vs. CP, pre-peaking or peaking;

† P<.01, pre-peaking vs. peaking.

Total annual HIT duration (including competitions) was 63±14 h (46–85 h) distributed across 106±20 sessions (85–147) throughout the year. The relative distribution of HIT duration in intensity zones 3, 4 and 5 was 39±10%, 37±13% and 24±13% respectively, and 32±6%, 38±14% and 30±13% according to a SG distribution. Monthly frequency of HIT sessions increased from GP to SP (*P*<.01). In addition, the monthly frequency of intensity zone 5 sessions increased from GP to SP and then remained unchanged in the CP (*P*<.01) (Figure 3, B). Weekly HIT patterns during each training phase are presented in Table 4.

### Peaking characteristics

**Figure 4:**
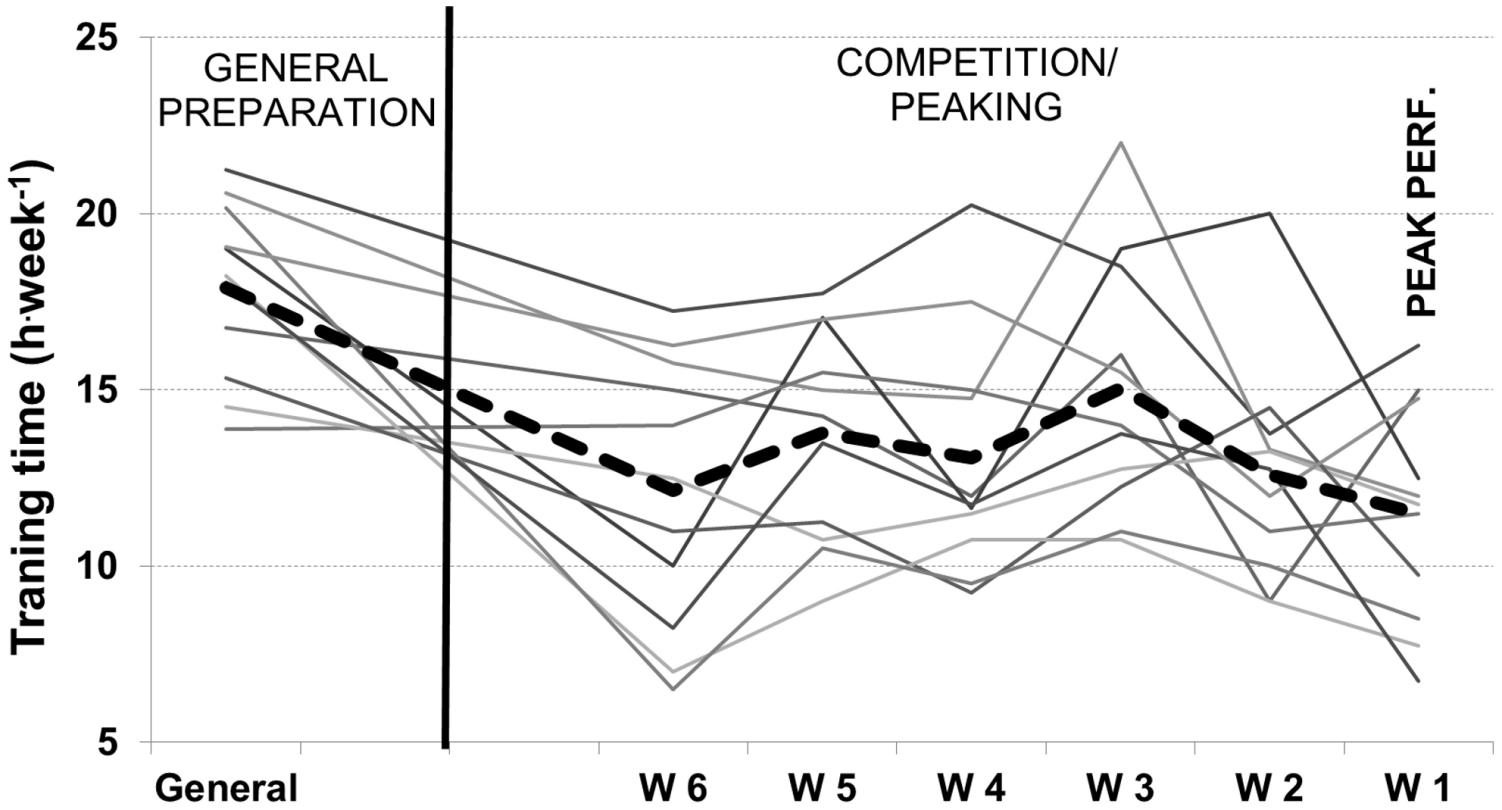
Individual peaking characteristics. Individual (lines) and average (dotted bold line) total weekly training volume during GP, and the last 6 weeks prior to championship title.

**Figure 5:**
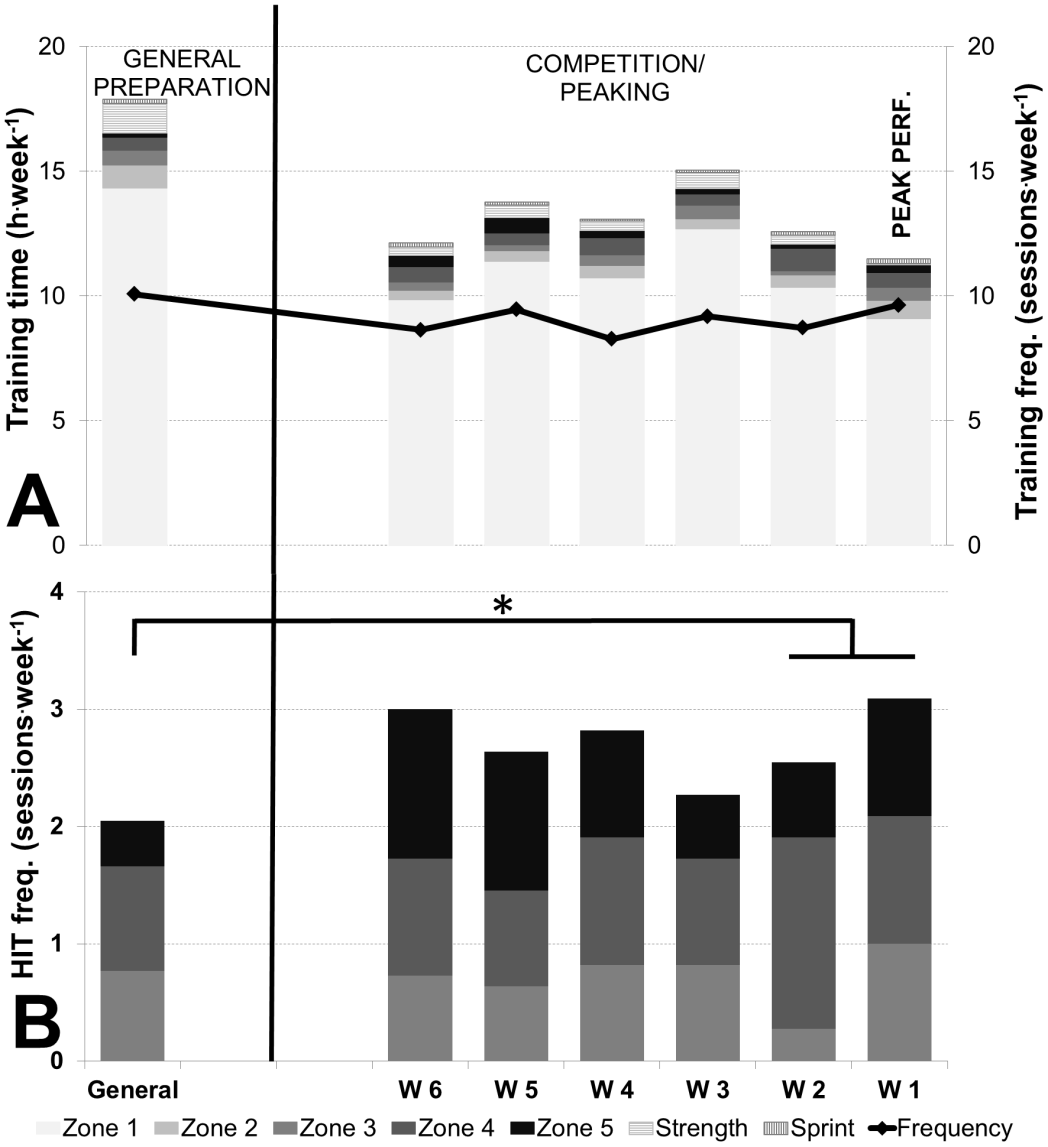
Peaking characteristics. A: Weekly training time (h) distributed into endurance training (zones 1–5), strength and sprints (bars, y-axis), and total training frequency (sessions) (line, z-axis) during GP, and during the last 6 weeks prior championship title. B: HIT frequency (sessions) distributed into zones 3, 4 and 5 (bars, y-axis) during GP, and during the last 6 weeks prior to championship title. There was a statistically significant difference (*P*<.05) in total HIT sessions and zones 3 and 5 respectively across GP, pre-peaking phase and peaking phase. Pairwise post-hoc tests showed: * Difference in total HIT sessions across phases (*P*<.01). There were no statistically significant differences in zones 3, 4 or 5 across phases.

Total training time (h.wk^−1^) decreased by 9±14% from the pre-peaking to peaking phase, but this did not reach statistical significance. However, the reduction from GP, when training volume was highest, to the peaking phase, was 32±15% (*P*<.01). This decrease in total training volume was entirely due to a reduction in non-sport-specific training. Individual data for each of the 11 athletes are presented in Figure 4. The decrease in training volume from GP and pre-peaking phase to the peaking phase was achieved via a reduction in both endurance and strength training, while sprint training time remained stable, although there was a tendency for sprint training time to increase slightly from the pre-peaking phase to the peaking phase. There were no significant changes in total session frequency per week between the peaking phase and any of the other phases (Figure 5 A and Table 4).

There was non-significant decrease of 9±15% in LIT endurance training (h.wk^−1^) from the pre-peaking phase to the peaking phase. However, LIT training volume decreased by 31±17% (*P*<.01) from GP to the peaking phase. In contrast, HIT time (h.wk^−1^) remained stable from both pre-peaking phase to the peaking phase and from GP to the peaking phase (Figure 5 A and Table 4).

LIT endurance session frequency decreased from GP to the peaking phase by 21±24% (*P* = .016) but remained stable from pre-peaking phase to the peaking phase. Weekly HIT session frequency increased by 40±27% (*P*<.01) from GP to the peaking phase, but remained stable from pre-peaking phase to the peaking phase (Figure 5 B). Training volume and frequency distribution among zones 3, 4 and 5 through the different phases are presented in Figure 5 B and Table 4.

## Discussion

To the authors' knowledge, this is the first study to connect accurate annual day-to-day training data to a specific peaking period in a group of athletes achieving ultimate international success in an endurance sport. The main findings of the present study are: 1) The annual training data for these Olympic and World champion XC skiers and biathletes conforms to previously reported training patterns amongst elite endurance athletes. 2) In contrast, peaking characteristics for these gold medalists did not conform to suggested best practice for tapering strategies in elite endurance athletes, as derived from partly experimental studies.

### Annual training characteristics

#### Training volume

High training volume has emerged as a key commonality in successful endurance training [20], [1]–[3], [25]. Athletes in the current study trained ∼800 h.year^−1^across ∼500 annual training sessions although there were individual differences. This finding is in line with previous studies reporting training volume in elite XC skiers [1], [16]–[17]. Muscular loading differences and stress associated with different activities probably explain why there is large variation in reported annual training volume across sports. For example, top international runners are reported to train “only” 500–600 h.year^−1^ [7]–[8] while a case study of an international level triathlete reports >1000 h.year^−1^ [23]. The current data show a tendency for developments in training patterns during the time period from 1985 to 2011, with a positive relationship between total training volume and year of championship title (r = .59, *P* = .055). Increased training volume appears to be mainly due to increased frequency of training sessions from 1985 to 2011, while average duration per training session has remained relatively stable at 1.7±0.2 h.

During the entire training year, 94% of all training *time* was executed as endurance training. However, strength and sprint training appear to play an important role in the training of XC skiers [45]. Strength training was carried out as general, specific or maximal, while sprints included both specific ski sprint-related exercises and jumps. Interestingly, ∼90% of all strength and sprint training was executed during the preparation period (PP). In practice, this means two to three strength and sprints sessions.week^−1^ in PP compared to one weekly session during CP, typically conducted at the end of endurance training sessions. The main underlying philosophy for these athletes was to build up a prescribed strength level during PP and then maintain this level during CP. Unfortunately, systematic strength testing documentation was not available for these athletes. We are therefore not able to verify whether strength characteristics of these athletes were stable during CP. However, previous research suggests that one bout of strength training per week is sufficient to maintain strength levels over shorter time frames [46].

#### Activity forms

During the entire training year, 64% (∼500 h) of all training was executed with sport-specific movement patterns (skiing/roller-skiing). However, over the course of the training year the amount of specific training increased from ∼50 to 90%. That is, in line with the early periodization models [48], when training load was highest in PP only ∼50% of all training was executed as ski or roller-ski. Otherwise, when training load was lowest in CP, >90% was performed as sport-specific training.

Sport-specific training is outlined as a key to improving 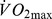 [51]–[52]. Hence, a high portion of sport-specific training during the CP for these athletes appears to be essential in order to reach an international performance level. However, we maintain that a large volume of non-specific activity forms during PP serve an important purpose in increasing trainability and improving general aerobic capacity [53]–[54].

#### Intensity distribution

Recently there has been some debate regarding findings suggesting that HIT induces superior physiological and performance adaptations compared with LIT [47]. The trend among endurance athletes is to adopt a polarized intensity distribution model integrating both intensity domains [7], [9], [16], [20], [25]. The present data consistently demonstrate that these 11 gold medalists executed a large proportion of their total training as LIT throughout the annual cycle. Total LIT time was progressively increased during PP, in line with some key features from the early periodization models of Matwejew [48], before being reduced dramatically during CP. However, it is important to emphasize that the marked intensity shift to more HIT described in Matwejew's models was not observed in this group of elite athletes.

The current study contributes unique knowledge to our understanding of the self-selected duration and distribution of HIT in elite endurance sports. Depending on the quantification methods used [43], results from several other studies suggest that an approximate 80/20% LIT/HIT distribution is optimal, although the percentage of HIT varies from ∼10–30% [7]–[9], [14]–[16], [44], [49]–[50] using a time in training zone method [43]. However, in the current study only 9% of annual endurance training time, or ∼60 h/∼100 sessions were reported to be above LT^1^. This is in contrast to other top Olympic athletes reported to perform a greater amount of HIT in addition to high total training volume [23], [28]–[29]. The total volume of HIT training was evenly distributed throughout the year with an average of 5±2 h or 9±3 sessions^.^month^−1^. Interestingly, it was also found that HIT training sessions were distributed virtually equally among zones 3, 4 & 5, with average durations of 0.8/0.6/0.5 h in zones 3/4/5 respectively. However, from the PP to CP, both duration and frequency in zones 3 and 4 were maintained, while the frequency of zone 5 training sessions increased. That is, as the main performance peak came closer, LIT time decreased dramatically while HIT patterns shifted towards a more polarized model, despite virtually constant HIT training time.

### Peaking practice

#### Training volume and specificity

Optimizing the reduction in training load during the peaking phase is believed to be a key to optimal championship performance [6], [30]. Training load is described as a combination of training volume, intensity and frequency [27]. A meta-analysis conducted by Bosquet et al [5] concluded that athletes could maximize taper-associated benefits by reducing training volume by ∼50%, without reducing training frequency or training intensity.

**Figure 6:**
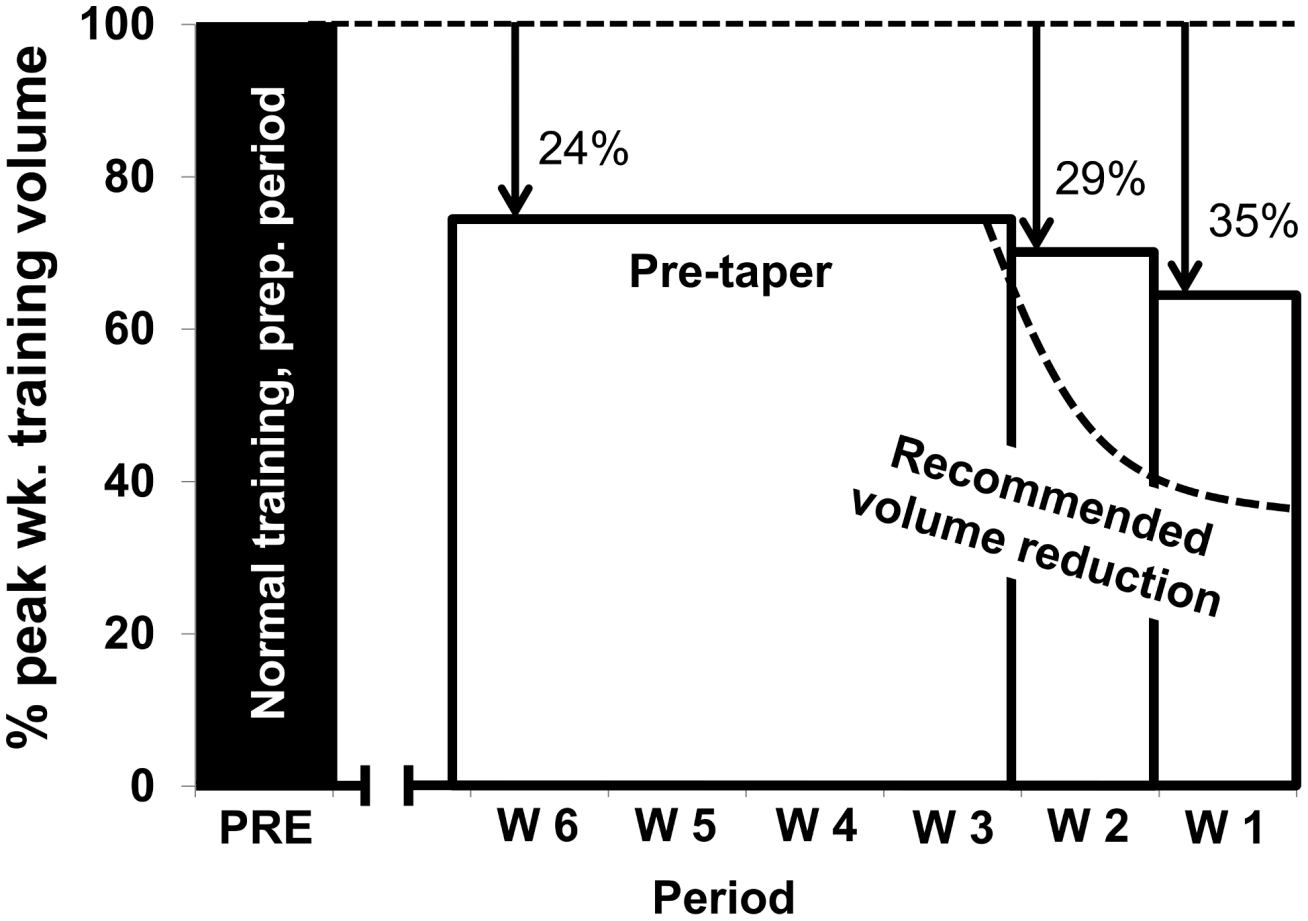
Taper comparison. Schematic representation of the actual taper observed in current study compared to recommended volume reduction. Adapted from Mujika & Padilla [4].

In line with current best practice [4]–[6], we defined the peaking phase as the last 14 days prior to the athletes' most successful competition (Olympic/World Championship gold medal), and compared training patterns in this final training phase to the penultimate phase beginning 4 weeks prior to the peaking phase (pre-peaking phase). With regards to training volume, we found only a 4 and 15% (NS) decrease in training volume during days -14 to -8 and days -7 to-1 respectively, compared to the pre-peaking phase. This is substantially less than the current taper recommendations of a ∼50% reduction (Figure 6). It is possible to speculate as to why these champion athletes chose a strategy very different from experimentally derived optimum. Bosquet et al [5] reported no effect on performance if the reduction in training volume was 20% or less. However, there was large individual variation in peaking behavior in the current study, and no clear patterns emerged. In fact, 4 of the 11 athletes increased their training volume during the last seven days. However, existing research has limitations in terms of narrow focus on one single competition [32]. In contrast, our results demonstrate that competitions are frequently integrated into the peaking process in elite sports. The competition schedule, designed by the International Ski Federation, is crucial in planning a taper and must be integrated into the peaking regime. The WC season in these sports typically consists of two competition days per week over up to 14 weeks with a maximum of two to four competition free weeks. Such a schedule may interfere with an optimal tapering process. Rather than incorporating a single tapering phase, such a schedule may rather require the athlete to perform repeated “mini-tapers” prior to each competition.

Since there was minimal decrease (NS) in overall training volume during the four-week pre-peaking period, we chose to compare training performed during the peaking phase to GP, where weekly training volume was highest. Once the athletes started their WC season, in either XC or biathlon, their total training volume was consistently lower than that reported during GP. Relative to GP, training volume was, respectively, 29 and 35% lower during the penultimate and final weeks before each athlete's gold medal race. High competition stress load and frequent travel may dictate the reduced training volume during this phase, rather than a predetermined periodization model. These data indicate that peak training volume for these athletes was markedly dissociated in time from peak performance by up to 4 months, even accepting individual variations. It is unclear whether high training volume executed during PP 4–9 months prior still influences physical capacity during the peaking phase, following an extended period of reduced training volume where competitions themselves become a key source of HIT.

Several decades ago, Hickson et al [55] reported that trained athletes retain most of their physiological and endurance performance adaptations during 15 subsequent weeks of reduced training. However, for an Olympic athlete, even a small performance decrement associated with reduced training could be the difference between a medal and fourth place. Unfortunately, similar to strength performance, we do not have data for endurance tests throughout the year. Our objective testing data for these athletes terminates 3–4 months prior to their gold medal performances. In elite practice, laboratory testing typically ends when the competitive season begins. However, in a similar group of athletes with virtually identical training patterns as in the current study, Losnegaard et al [17] found that aerobic physiological adaptations were maintained, and performance and anaerobic adaptations were even enhanced, several months after peak training volume.

To our knowledge, no data are available providing mechanistic links that span such an extended time period. It is possible to speculate that a prolonged period of high training volume during PP could favorably alter genomic sensitivity to training during the season through epigenetic mechanisms [51]. Such cellular level adaptations to high training volume could be a mechanistic bridge linking PP training characteristics to training effects several months later, when high training volumes are precluded by the competition and travel stress load.

During both the pre-peaking phase and the peaking phase, virtually all (92%) training was conducted as XC skiing. This shift towards more specific movement patterns when competition approaches may explain why peak performance is possible even after several months with reduced training volume [51]–[52].

#### Training frequency

The athletes in the current study trained, on average, 8–10 sessions^.^week^−1^, with no significant differences in training frequency between the peaking phase and other phases (Table 2). This finding is in line with current taper recommendations [4]–[6]. Nor were there any significant differences in the number of LIT or HIT sessions from the pre-peaking phase to the peaking phase. However, LIT frequency decreased from GP (8 sessions^.^week^−1^) to the peaking phase (6 sessions^.^week^−1^), indicating that the observed reduction in total LIT time was a result of both reduced session frequency and session duration.

#### Intensity distribution and rest days

Adaptive stimuli from HIT sessions appear to be a key component in maintaining and enhancing physiological and performance adaptations q during a taper period [36]–[37], [39]. McNeely & Sandler [31] reported that frequent short HIT bouts >90% 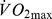 are more effective than LIT to enhance endurance performance, and that, during a taper, steady-state workouts should be replaced by HIT intervals and short sprints in order to improve performance. Interestingly, we found that HIT duration did not change (1.3 h^.^week^−1^) during any of the phases. However, HIT frequency increased from 2 sessions^.^week^−1^ in GP to 3 sessions^.^week^−1^ in the peaking phase (*P*<.01). In addition, there was a tendency towards increased sprint training duration from the pre-peaking phase to the peaking phase. Hence, HIT sessions during the peaking phase were typically executed more frequently but with shorter duration than during GP, alongside more frequent bouts of sport-specific “anaerobic sprint training”. Examining distribution of training among intensity zones 3, 4 and 5, we observed a tendency toward a decrease in zones 3 and 4 and an increase in zone 5 in both duration and frequency from GP to CP. This suggests that total HIT duration did not change throughout the year, but that the actual executed intensity shifted towards a more polarized model as the major competition approached.

**Figure 7:**
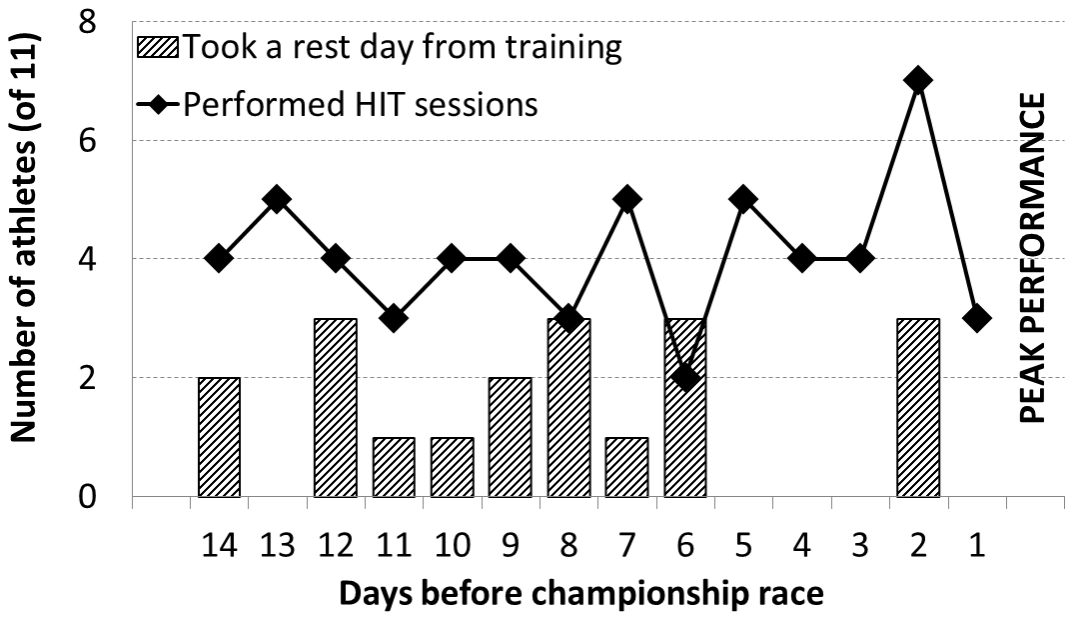
Peaking phase. Number of athletes (of 11) who performed HIT sessions (line) and number of athletes who took a rest day from training (bars) during the final 14 days prior to peak performance.

To our knowledge, details regarding best practice models of HIT patterns and recovery strategies during the final days prior to peak performance are lacking in the literature. However, the current data show that short bouts of HIT were performed evenly throughout the final 14 days (∼5 sessions in total per athlete) (Figure 7). Interestingly, 10 out of 11 athletes performed a HIT session within 48 h of competition. The exact intensity during these HIT sessions is somewhat inconsistent, but was typically above LT^2^. Competitions performed during the final days but not seen as “primary events” were also integrated into the peaking strategy. Whether these contribute to a beneficial peaking regime, or interfere with the optimal strategy is not clear. Eight of 11 athletes in the present study competed in at least one championship final prior to the event in which they won a gold medal. With regard to recovery strategies, rest days were typically concentrated in days 12 to 6. Among all 11 athletes, only 3 athletes took a rest day during the last 5 days, compared with 14 athlete rest days taken in the middle period of the peaking phase. That is, rest days were 3 times more likely to be taken during the middle portion of the peaking phase (days 12–6) compared with the final 5 days. However, it is not clear whether this organization of HIT and rest days during the final 14 days was the result of strategic planning to optimize performance, or merely coincidental. It has been previously reported that runners taking a rest every third day during a six day taper performed worse than those athletes who trained every day [56], and this topic may be a fruitful area for future research.

#### Altitude training

Altitude training is incorporated into the training of most world-class XC skiers, and is a consistent feature of Norwegian endurance training. For athletes in the current study, precise records are not available regarding all days spent at altitude or the specific altitude at which each training session was performed. For the last 2–3 decades, 4–6 annual training camps of 14–21 d duration living at 1800–2000 m above sea level and training at 1200 to 2800 m above sea level, have been integrated throughout the annual cycle. The aim of these altitude training camps is to stimulate increased hemoglobin mass, and specifically acclimate to competition venues located above 1400 m. The athletes in the current study typically spent 60 to 100 days training at altitude during the season quantified, although this was likely somewhat lower for those athletes winning gold prior to 1992. In addition, where championship events were held at moderate altitude (e.g. in Salt Lake City, 2002) altitude camps were also an important feature of the final weeks of training. Based on a previous study of 29 XC skiers training at altitude [43], objective data suggest that intensity distribution during altitude camps shifts towards lower intensity. Training at the highest aerobic intensities during such camps is essentially absent, unless it is performed at reduced altitudes. The likely impact of this emphasis on altitude training was to somewhat reduce the amount of HIT performed during PP.

Winning an international title in endurance sports clearly requires outstanding physiological capacity and performance level. Controlled laboratory trials of world-class elite athletes are challenging, and training literature based on less well-trained individuals may be misleading when linking findings to elite athletes. Our current data outlines unique and accurate day-to-day training data throughout a season that concluded with each athlete winning an Olympic or World championship title. Experimental approaches may in many ways be artificial, while descriptive training studies allow investigation of elite endurance athletes in a real-life situation. This may therefore provide a fruitful foundation from which to generate novel experimental research questions.

We did not find evidence of athletes following the current tapering recommendations regarding training volume reduction. However, when comparing training patterns during the peaking phase to training executed during PP several months earlier, we found a picture more analogous to that derived from experimental studies, although the magnitude of training time reduction was still lower. It is possible to speculate as to whether the medal-winning performances of these athletes was truly representative of their best possible performance, or if they could have skied even faster had they followed recommended tapering strategies specifically for that one event. On the other hand, the more progressive reduction in training time from GP to CP observed in the current study, continued to a lesser degree throughout the CP up until the major competition, may be the ideal strategy in sports where the competition schedule is organized as it is in XC skiing and biathlon. A three month competition phase during which athletes are typically required to compete once or twice every week, precludes the application of the recommended tapering strategy presented in the research literature. Regardless, the performance of these athletes was sufficient to beat the rest of the field on the day, and take home the gold medal.

A central concern in a descriptive study such as this, where training self-report is the key data source, is whether the data are accurate and valid. We have recently demonstrated that elite endurance athletes report their training accurately, although we found some small discrepancies related to intensity distribution [57]. We believe the current data represent the same validity as shown in Sylta et al [57], since both athlete groups used similar monitoring routines, and some of the athletes are, in fact, represented in both papers. In addition, athletes recorded their training on a daily basis, which likely reduced reporting error.

## Conclusions

These data show that winning an international title in XC skiing or biathlon requires a training load of ∼800 h/500 sessions^.^year^−1^, of which ∼500 h is executed as sport-specific movement patterns. Endurance training time for these athletes was distributed as approximately 90% LIT and 10% HIT, equal to a ∼80/20% SG distribution. Training volume was highest during GP and decreased progressively during SP and CP. Concurrently, the proportion of sport-specific training increased markedly. Total amount of HIT remained stable across all phases, although HIT training patterns tended to become more polarized in CP.

These athletes did not appear to incorporate a taper in the final weeks leading up to competition, with training volume, frequency and intensity remaining unchanged from the pre-peaking phase to the peaking phase. Hence, we did not observe the recommended ∼50% training volume reduction that has been proposed as the optimal tapering strategy based on previous experimental studies. However, there was a clear reduction in training volume from GP to the peaking phase. This reduction was almost entirely due to a reduction in non-sport-specific LIT with virtually all training during the pre-peaking phase and the peaking phase composed of ski training. Only three out of 11 athletes incorporated a rest day in the final five days leading up to the best athletic performance of their career, A very large training load during the GP appears to be an important precondition for exceptional athletic performance several months later, although exactly how training loads in June-October are mechanistically connected to performance several months later remains unclear.
